# Composition, diversity and function of intestinal microbiota in pacific white shrimp (*Litopenaeus vannamei*) at different culture stages

**DOI:** 10.7717/peerj.3986

**Published:** 2017-11-06

**Authors:** Shenzheng Zeng, Zhijian Huang, Dongwei Hou, Jian Liu, Shaoping Weng, Jianguo He

**Affiliations:** 1 State Key Laboratory of Biocontrol, Guangdong Provincial Key Laboratory of Marine Resources and Coastal Engineering, School of Marine Sciences, Sun Yat-sen University, Guangzhou, China; 2 School of Life Sciences, Sun Yat-sen University, Guangzhou, China

**Keywords:** Intestinal microbiota, Microbial composition, Pacific white shrimp, Diversity, Function, Culture stage

## Abstract

Intestinal microbiota is an integral component of the host and plays important roles in host health. The pacific white shrimp is one of the most profitable aquaculture species commercialized in the world market with the largest production in shrimp consumption. Many studies revealed that the intestinal microbiota shifted significantly during host development in other aquaculture animals. In the present study, 22 shrimp samples were collected every 15 days from larval stage (15 day post-hatching, dph) to adult stage (75 dph) to investigate the intestinal microbiota at different culture stages by targeting the V4 region of 16S rRNA gene, and the microbial function prediction was conducted by PICRUSt. The operational taxonomic unit (OTU) was assigned at 97% sequence identity. A total of 2,496 OTUs were obtained, ranging from 585 to 1,239 in each sample. Forty-three phyla were identified due to the classifiable sequence. The most abundant phyla were Proteobacteria, Cyanobacteria, Tenericutes, Fusobacteria, Firmicutes, Verrucomicrobia, Bacteroidetes, Planctomycetes, Actinobacteria and Chloroflexi. OTUs belonged to 289 genera and the most abundant genera were *Candidatus_Xiphinematobacter*, *Propionigenium*, *Synechococcus*, *Shewanella* and *Cetobacterium*. Fifty-nine OTUs were detected in all samples, which were considered as the major microbes in intestine of shrimp. The intestinal microbiota was enriched with functional potentials that were related to transporters, ABC transporters, DNA repair and recombination proteins, two component system, secretion system, bacterial motility proteins, purine metabolism and ribosome. All the results showed that the intestinal microbial composition, diversity and functions varied significantly at different culture stages, which indicated that shrimp intestinal microbiota depended on culture stages. These findings provided new evidence on intestinal microorganism microecology and greatly enhanced our understanding of stage-specific community in the shrimp intestinal ecosystem.

## Introduction

Intestinal microbiota is a complex organ ecosystem with multiple functions critical for host health (Al-Harbi & Uddin, 2005; Ramirez & Romero, 2017). It has been reported that the stable intestinal microbiota influences myriad host functions like the establishment of microorganisms in the intestinal tract and infectious susceptibility (Wu et al., 2010; Ravel et al., 2014). During the host development, different shifts happen in intestinal microbiota depending on host age (Fraune & Bosch, 2010; Li et al., 2017). In an effort to better understand the relationship between intestinal microbiota and host, it is necessary to identify the composition of the microbiota and understand how they vary during the host development.

The pacific white shrimp, *Litopenaeus vannamei*, is becoming increasingly important for aquaculture as one of the most profitable species in shrimp farming, with the production being more than 3 million tons per year (Zhang et al., 2016). In recent years, some bacterial diseases in shrimp, such as early mortality syndrome (EMS), acute hepatopancreatic necrosis disease (AHPND) and hepatopancreas necrosis syndrome (HPNS), have led to the shrimp production dropped to nearly 60% and caused global losses to the shrimp farming industry estimated at more than $1 billion per year (Lightner et al., 2012; Lee et al., 2015; Huang et al., 2016). Some previous studies reveal that many bacterial diseases are associated with the shifts and imbalance of intestine microbiota in other aquaculture animals (Perez et al., 2010; Li et al., 2016) and the probiotic addition is helpful for maintaining the intestinal bacterial balance (Irianto & Austin, 2002; Balcazar et al., 2006).

Some studies have been conducted on intestinal microbiota in aquaculture animals, such as grass carp (Wu et al., 2012; Li et al., 2015), yellow catfish (Wu et al., 2010) and atlantic cod (Dhanasiri et al., 2011). The intestinal microbiota of pacific blue shrimp and black tiger shrimp have been well investigated (Rungrassamee et al., 2014; Cardona et al., 2016), while most reports about pacific white shrimp focus on the microbial community of the surrounding water (Tang et al., 2014; Hou et al., 2016) and the effect of diet on intestinal microbiota (Zhang et al., 2014). A previous report shows that the shift of microbial composition and structure is less affected by the surrounding environment than by the host development (Li et al., 2017), and fish intestinal microbiota is mainly shaped by intestinal environment and some changes accompanying the host development (Yan et al., 2016). Knowledge of the intestinal microbiota of pacific white shrimp at different culture stages is still limited.

The functional potential of microbial community reflects the connection between intestinal microbiota and the surrounding environment (Abubucker et al., 2012). Therefore, the functional characterization of the microbial community is necessary to determine microbial function in the intestine. In other animals, the microbial functions have been well studied, such as grass carp (Wu et al., 2015) and fine flounder (Ramirez & Romero, 2017). However, the function of shrimp intestine microbiota has not been extensively explored yet.

Some conventional methods had been adopted to study the microbiota, including culture-dependent plate counting method (Tuyub Tzuc et al., 2014), clone libraries (Wu et al., 2010) and polymerase chain reaction-denaturing gradient gel electrophoresis (PCR-DGGE) (Dhanasiri et al., 2011). However, the above traditional methods were certainly limited since it would underestimate the overall diversity and it was difficult to profile a comprehensive community in complex environments. The high throughput sequencing, which can generate more reliable and sufficient information through the amplification and identification of 16S rRNA gene, can provide a profile of the whole community (Glenn, 2011; Sun et al., 2014). Many studies detected the intestinal microbiota by high throughput sequencing to obtain a high-resolution map of the intestinal microbiota in other aquaculture animals (Al-Harbi & Uddin, 2005; Wu et al., 2012; Rungrassamee et al., 2014; Ramirez & Romero, 2017).

This study aimed to evaluate the difference of intestinal microbiota at different culture stages. The present study compared the composition, diversity and functions of intestinal microbiota in pacific white shrimp, which showed that intestinal microbiota varied significantly at different culture stages. This study greatly enhanced our understanding of stage-specific community assembly patterns in the shrimp intestine microecosystem.

## Materials and Methods

### Sample collection

From July to October 2015, 22 intestine samples were collected from 5 shrimp ponds in a commercial shrimp farm, Maoming, Guangdong, China (21.68°N, 110.88°E). Healthy shrimp were collected every 15 days from the larval stage (stage1, 15 dph) to adult stage (stage 5, 75 dph) (Table S1).

Each pond was approximately 2,600 m^2^ and the average depth was 1.5 m. Shrimp larvae with average length of 0.7 cm were cultured at a stocking density of 200,000 shrimps each pond. The water temperature was relatively stable at approximately 32 °C. The pH value ranged from 7.5 to 8.61. The concentration of NH_3_-N, NO_2_-N, NO_3_-N, PO${}_{4}^{3-}$ and SO${}_{4}^{2-}$ were in range of 0.0089∼1.1095 mg L^−1^, 0.0022∼0.9869 mg L^−1^, 0.0323∼3.3007 mg L^−1^, 0.0171∼0.3131 mg L^−1^ and 0.0012∼0.3777 mg L^−1^. There was no antibiotic application during the culture period. Some probiotics, including *Lactobacillus* and *Bacillus* from Guangdong Zhongtai Biology Co., Ltd. (Guangdong, China), have been mixed with feed and applied to ponds once a week.

Sampling was according to the previously reported methods (Oxley et al., 2002; Rungrassamee et al., 2014). The shrimp’s surface was sterilized with 70% ethanol and the intestine was aseptically dissected. The intestine was put into a 2 mL centrifuge tube which contained sterile glass beads and 1.5 mL PBS buffer. The tube was thoroughly vortexed for 3 min and centrifuged at 10,000 g for 1 min. Samples were immediately stored at −80 °C before DNA extraction.

### DNA extraction and sequencing

Total DNA was extracted by the PowerFecal DNA Isolation Kit (MoBio, Palo Alto, CA, USA) following the manufacturer’s directions. The concentration and purity of total DNA were determined by NanoVuePlus Spectrophotometer (GE Healthcare, USA) and 1% agarose gels. The primer pair 515F (5′-GTGCCAGCMGCCGCGGTAA-3′) and 806R (5′-GGACTACHVGGGTWTCTAAT-3′) were used to amplify the V4 hypervariable region of 16S rRNA gene, which was modified with a barcode tag with a random 6-base oligos (Bates et al., 2011). Sequencing libraries were generated via using TruSeq DNA PCR-Free Sample Preparation Kit (Illumina, San Diego, CA, USA). In addition, the library quantity was assessed on Qubit 2.0 Fluorometer (Thermo Scientific, Waltham, MA, USA). The libraries were sent for sequencing by Illumina Hiseq2500 platform (Illumina, San Diego, CA, USA), which was conducted by Novogene Bioinformatics Technology Co.,Ltd. (Beijing, China). Raw data generated from Hiseq2500 platform were paired-end reads.

### Data analysis

Based on the unique barcode, sequences were assigned to samples and then removed off the barcode and primer sequence by QIIME (Version 1.7.0, http://qiime.org/index.html) (Caporaso et al., 2010). In order to merge paired-end reads when at least some of the reads overlap the read generated from the opposite end of the same DNA fragment, FLASH (Version 1.2.7, http://ccb.jhu.edu/software/FLASH/) was used to get raw tags (Magoc & Salzberg, 2011). In terms of quality control, raw tags with low quality (quality value  ≤19, homopolymers  ≥3 bases and sequence length ≤200 bp) were filtered by QIIME according to the QIIME quality filtering process in a bid to obtain the high-quality clean tags. Tags were compared with Gold database (http://drive5.com/uchime/uchime_download.html) by UCHIME algorithm (http://www.drive5.com/usearch/manual/uchime_algo.html) so as to remove off chimera sequences and then the effective tags were finally gained (Edgar et al., 2011).

Sequences with over 97% similarity were considered as the same OTUs for further annotation (Edgar, 2013). To align the sequences, the GreenGene Database (http://greengenes.lbl.gov/download) was used as a reference database (DeSantis et al., 2006). Later, the taxonomic information was annotated by RDP classifier (Version 2.2, http://sourceforge.net/projects/rdp-classifier/) with 80% confidence threshold. OTUs abundance information was normalized using a standard of sequence number corresponding to the sample with the least sequences. The Venn diagram, which was used to find out the shared OTUs among groups, was conducted by Draw Veen Diagram online tool (http://bioinformatics.psb.ugent.be/webtools/Venn/). Alpha diversity, showing the complexity of species for one sample through 5 indices, including Chao, Shannon, Simpson, ACE and Good’s coverage, was calculated by QIIME following the tutorial (http://qiime.org/scripts/alpha_diversity.html) and displayed via R software (Version 2.15.3). Beta diversity, used to evaluate differences of samples in species complexity, was calculated by QIIME (http://qiime.org/scripts/beta_diversity.html). Unweighted pair-group method with arithmetic means (UPGMA) was conducted to report the hierarchical clustering of samples by QIIME following the guidance (http://qiime.org/scripts/jackknifed_beta_diversity.html). Statistical analyses of alpha diversity were calculated by analysis of variance (ANOVA) to compare the significant differences at different culture stages by SPSS (Version 21). Multiple-response permutation procedure (MRPP) was conducted to test significant difference between any two of compared culture stages using the vegan package in R (Cai, 2006). Permutational analysis of multivariate dispersions (PERMDISP) was used to test whether the microbial community varied at different culture stages by using the vegan package in R (Anderson, 2006). Permutational multivariate analysis of variance (PerMANOVA) was conduct to compare microbial composition and function dissimilarities (Anderson, 2001). A calculated *P* value < 0.05 was considered to be statistically significant.

### Microbial function prediction based on 16S rDNA data

The OTU table was used to generate the inferred metagenomic data by using PICRUSt (version 1.1.0) to predict the metagenomic functional capacity (Langille et al., 2013). The abundance values of each OTU were firstly normalized to its 16S rRNA copy number respectively. Predicted functional pathways were annotated by using the Kyoto Encyclopedia of Genes and Genomes (KEGG) (Kanehisa et al., 2012) at level 2 and level 3 KEGG orthology groups (KOs) (Langille et al., 2013). The accuracy of the predictions of the metagenomes was assessed by computing the nearest sequenced taxon index (NSTI). The associated metabolic pathways were identified by means of employing the HMP unified metabolic analysis network (HUMAnN) (Abubucker et al., 2012). Moreover, the KOs at different culture stages were further examined by PerMANOVA. The relationships among functional capacities were analyzed by principal component analysis (PCA).

### Accession number

The raw data in this study have been deposited in the GenBank Sequence Read Archive database. The accession number is SRX2946975.

## Results

### Composition of intestinal microbiota

Quality and chimera filtration of the raw data produced totally 1,408,105 high quality sequencing reads from 22 samples, belonging to five culture stages, with an average of 64,005 reads, ranging from 41,250 to 79,515 (Table 1). By performing the alignment at an average length of 253 bp, OTUs were clustered at 3% distances, among which each OTU represented a unique phylotype. Finally, 2,496 OTUs were obtained and the number of OTUs detected in each sample ranged from 585 to 1,239, with an average of 880 OTUs (Table 1).

**Table 1:** Sequencing and OTU classification information. Summary of sequencing read analysis, numbers of OTUs, and numbers of OTUs that can be classified into different levels (phylum, class, order, family and genus). A, B, C, D and E stand for the ponds. 1, 2, 3, 4 and 5 stand for the culture stages.

Sample	Trimmed tags	OTUs	Phylum	Class	Order	Family	Genus
A1	73,996	645	18	46	78	103	73
B1	64,306	591	20	48	84	109	83
C1	64,282	817	21	54	100	131	115
D1	66,164	622	14	41	69	95	69
E1	70,365	737	17	44	70	97	77
A2	61,460	1,169	32	74	121	161	147
D2	70,166	1,170	26	65	105	140	132
E2	63,705	910	18	51	94	105	84
A3	61,660	976	28	64	112	134	108
B3	62,061	971	25	59	101	132	117
C3	38,103	993	31	72	120	153	127
D3	69,588	1,150	27	63	113	154	147
E3	60,883	947	21	50	91	112	91
A4	45,440	978	29	66	101	134	132
B4	38,348	585	22	48	85	109	84
C4	40,220	643	19	46	77	101	83
D4	62,236	722	18	47	81	105	98
E4	61,853	708	18	46	77	100	86
A5	57,143	1,075	28	68	111	143	122
C5	66,736	1,239	27	66	111	156	143
D5	68,803	757	17	40	74	104	87
E5	67,102	972	25	53	90	114	90

**Figure 1:**
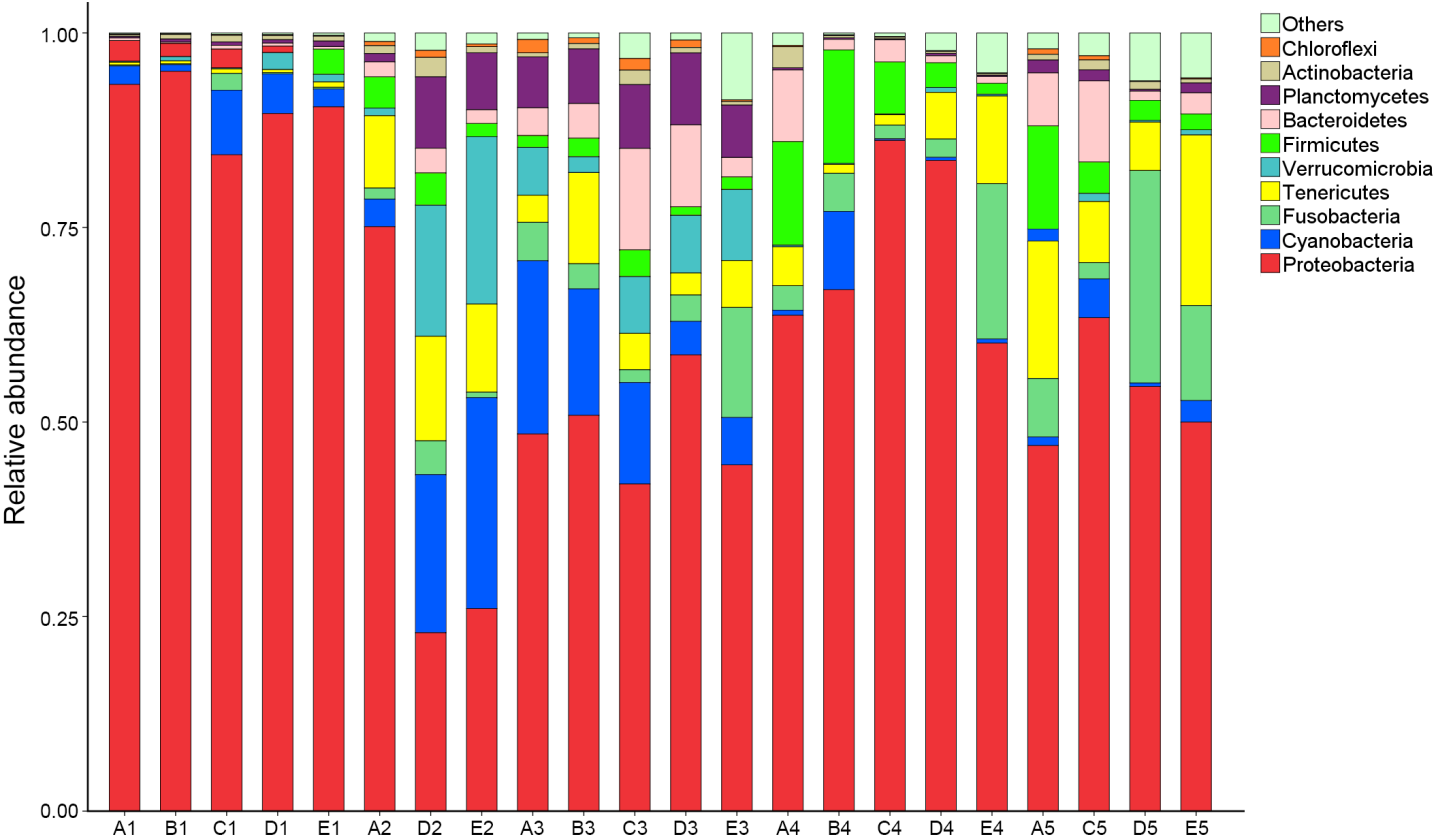
Relative read abundance of different bacterial phyla within the different communities. Sequences that cannot be classified into any known group are assigned as ‘Others’ bacteria. A, B, C, D and E stand for the ponds. 1, 2, 3, 4 and 5 stand for the culture stages.

OTUs were identified into 43 phyla. Sequences that could not be classified into any known groups were assigned as ‘others’. The most relative abundant phyla in all samples were Proteobacteria (63.5%), Cyanobacteria (7.0%), Tenericutes (6.5%), Fusobacteria (5.3%), Firmicutes (4.1%), Verrucomicrobia (3.6%), Bacteroidetes (3.6%), Planctomycetes (2.9%), Actinobacteria (0.8%) and Chloroflexi (0.4%) (Fig. 1). Proteobacteria was the most abundant phylum among 21 samples except sample E2, in which Cyanobacteria was the most abundant phyla (27.8% relatively abundance).

**Figure 2:**
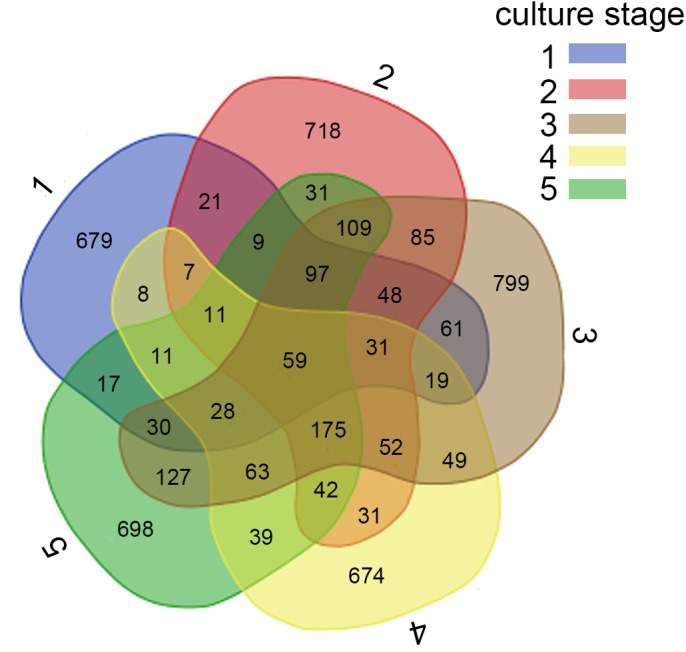
Analysis of the shared OTUs in different libraries. Venn diagram shows the unique and shared OTUs in the different libraries. A, B, C, D and E stand for the ponds. 1, 2, 3, 4 and 5 stand for the culture stages.

At genus level, a total of 289 taxa were identified. The top 10 genera were *Candidatus_Xiphinematobacter* (3.4%), *Propionigenium* (3.4%), *Synechococcus* (2.7%), *Shewanella* (1.3%), *Cetobacterium* (1.1%), *Bacillus* (0.9%), *Robiginitalea* (0.7%), *Fusibacter* (0.5%) and *Arcobacter* (0.5%) (Fig. S1). The abundance of *Lactobacillus* and *Bdellovibrio* were 0.04% and 0.002% respectively.

For further investigation of the dominant microbiota that exists in all samples, Veen diagram was constructed to identify dominant OTUs presented in intestine (Fig. 2). There were 59 OTUs shared among 22 samples, representing 83.1% of the total reads. Among the shared OTUs, 17 OTUs (28.8%) belonged to Proteobacteria, 5 OTUs (8.5%) belonged to Cyanobacteria and 5 OTUs (8.5%) belonged to Bacteroidetes.

### Diversity, similarity and function analysis

The diversity and richness indices of all samples from five ponds were calculated in an effort to illustrate the complexity of each sample (Table S2). The completeness of sequencing was estimated with Good’s coverage, showing the probability of a randomly sequence already detected in the sample. The rarefaction curves approached the plateau (Fig. S2). The Good’s coverage ranged from 0.989 to 0.996, suggesting that additional 90 to 250 reads needed to be sequenced before discovering new OTUs. Shannon index and Simpson index were often used to quantify the diversity. The Shannon index ranged from to 1.936 to 6.592, while the Simpson index ranged from 0.273 to 0.968. The richness of each sample was calculated via Chao index and ACE index. Chao index ranged from 558 to 1,386, while ACE index ranged from 599 to 1,416.

**Figure 3:**
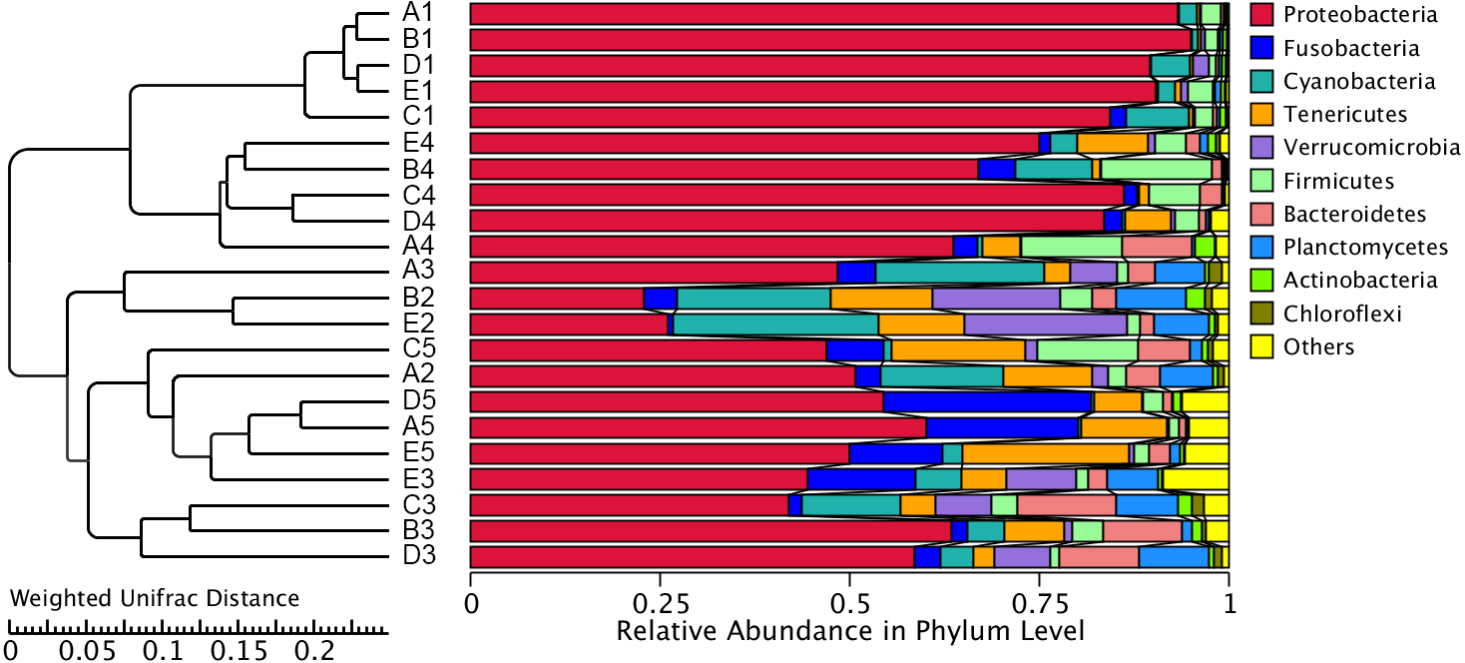
UPGMA clustering of samples. The UPGMA clustering was calculated with weighted Unifrac Distance. A, B, C, D and E stand for the ponds. 1, 2, 3, 4 and 5 stand for the culture stages.

**Figure 4:**
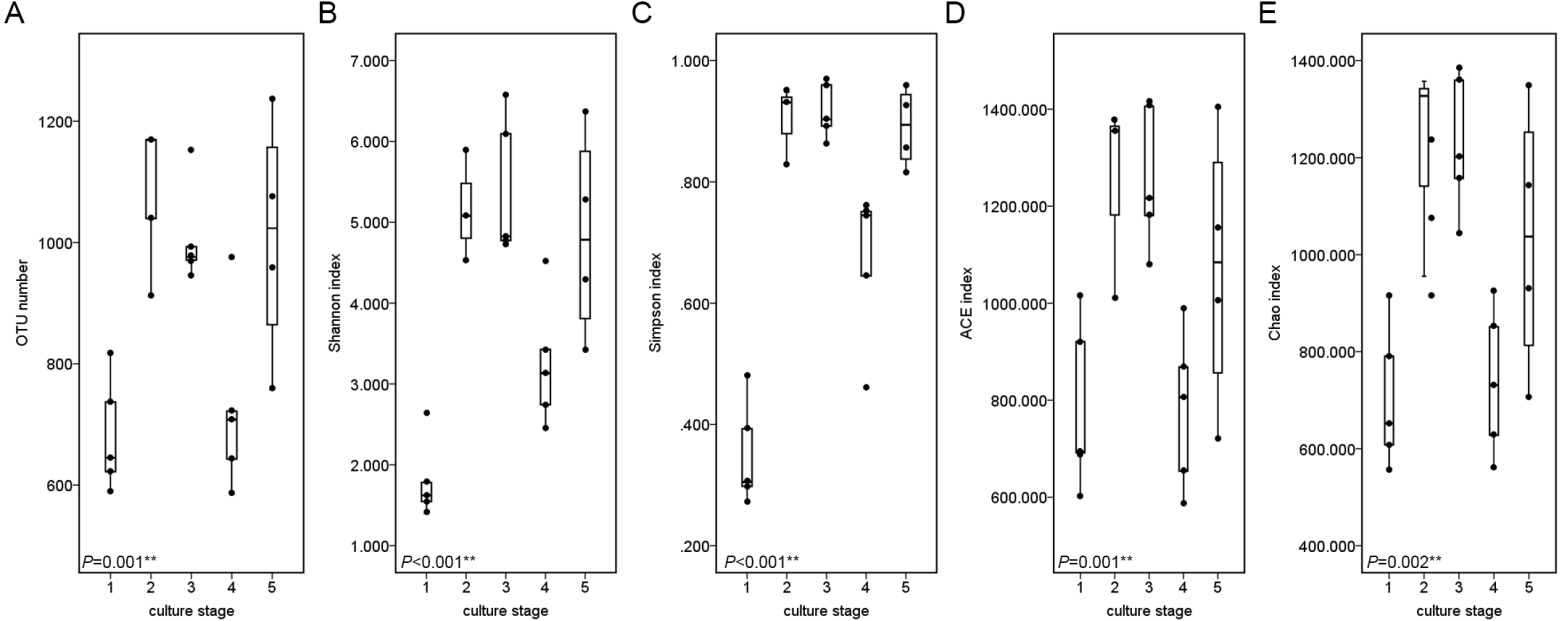
Boxplots figure of shows the range of different alpha diversity index. The Boxplots figure showed there was significant difference at different culture stages of OTU number (A), Shannon index (B), Simpson index (C), ACE index (D) and Chao index (E). The asterisk represents that there is significant difference in groups by ANOVA (*P* value < 0.05). The two-asterisk represents that there is extremely significant difference in groups (*P* value < 0.01).

**Figure 5:**
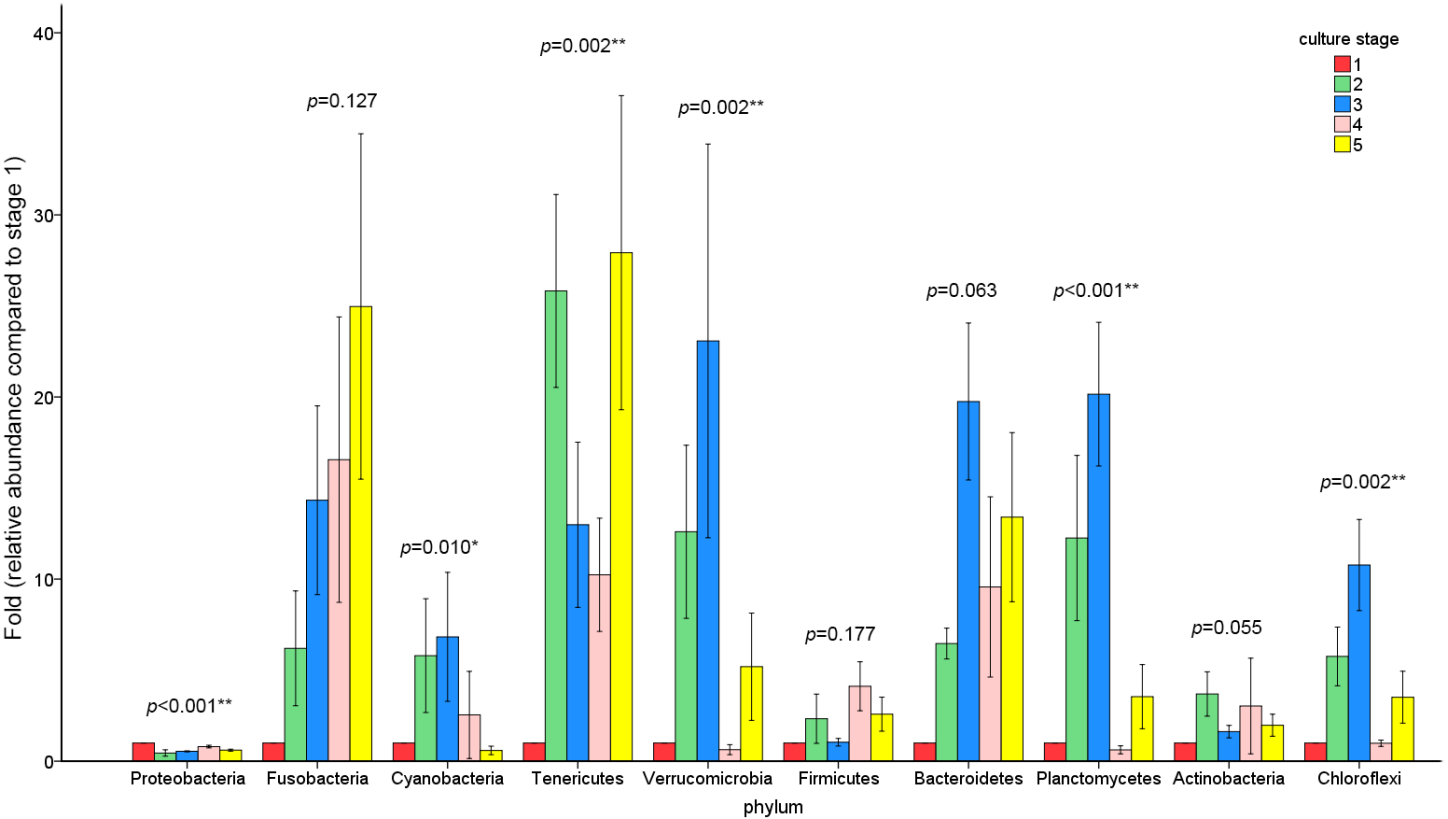
The abundance of the top 10 phyla at different culture stages. The relative abundance of each phyla at 5 culture stages is shown. The abundance of stage1 was given value 1 and the other stages were given the relative abundance compared to stage 1. The asterisk represents that there is significant difference in groups by PerMANOVA (*P* value < 0.05). The two-asterisk represents that there is extremely significant difference in groups (*P* value < 0.01).

The similarity and difference in different intestinal microbiota samples were further investigated. UPGMA clustering showed that almost all of the individual samples were clustered into groups according to the culture stage (Fig. 3). The OTU number, Shannon index, Simpson index, ACE index and Chao index were shown in boxplot graph (Fig. 4), and ANOVA showed that there was extremely significant difference in the OTU number, Shannon index, Simpson index, ACE index and Chao index at different culture stages (*P* value < 0.05) (Table 2). MRPP and PERMDISP showed the intestinal microbiota differed significantly between any two of compared stages (*P* value < 0.05) (Table 3). Among the top 10 phyla, PerMANOVA demonstrated the abundance of Proteobacteria, Fusobacteria, Tenericutes, Verrucomicrobia, Planctomycetes and Chloroflexi changed significantly at different culture stages (*P* value < 0.05) (Fig. 5).

### Functional prediction of the intestinal microbiota

**Figure 6:**
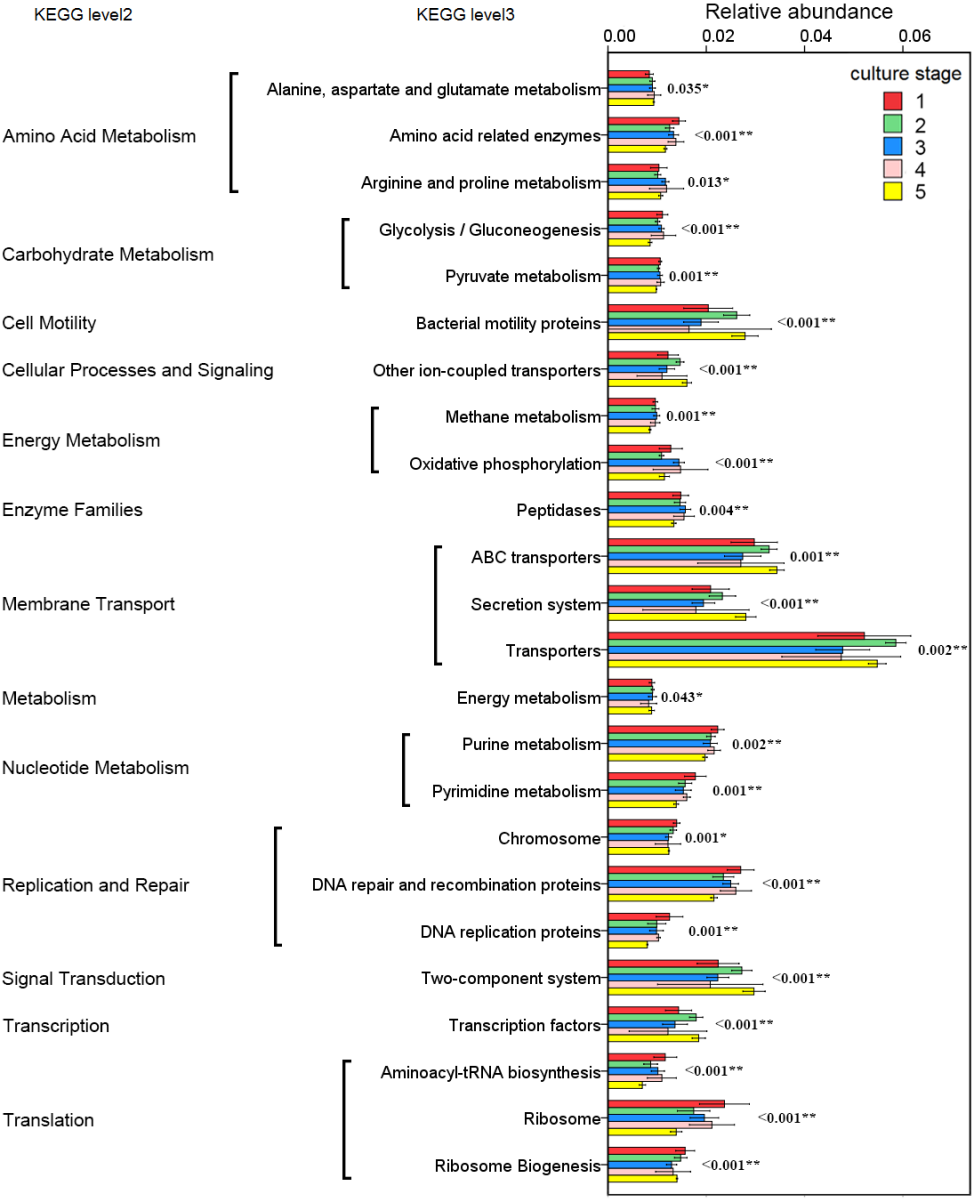
Predicted functions of the intestinal microbiota that varies significantly at different culture stages. The asterisk represents that there is significant difference in groups by PerMANOVA (*P* value < 0.05). The two-asterisk represents that there is extremely significant difference in groups (*P* value < 0.01).

**Figure 7:**
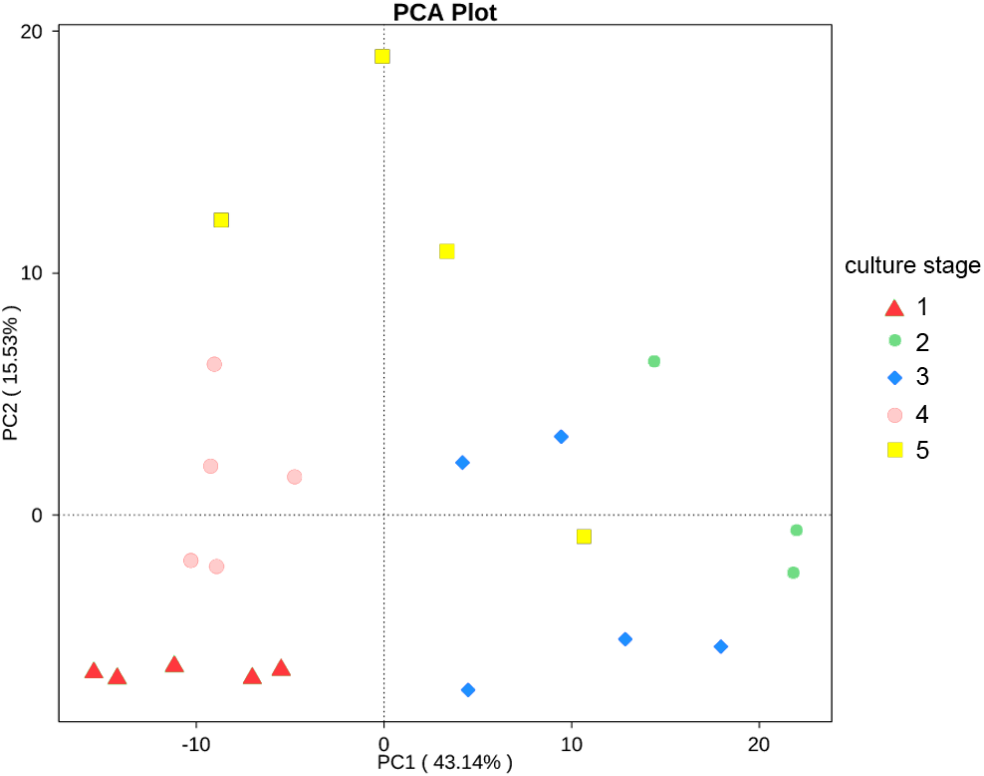
PCA shows the similarity of samples. PCA presents the similarity of KOs at different culture stages. Samples from the same culture stage were clustered closer.

The changes in the presumptive functions of the intestinal microbiota of pacific white shrimp were examined by predicting the metagenomes using PICRUSt. The accuracy of the prediction was evaluated by computing the NSTI, and the mean of the samples was 0.171 ± 0.023. The metagenomic prediction showed the intestinal microbiota was enriched with functions that were related to transporters, ATP-binding cassette (ABC) transporters, DNA repair and recombination proteins, two component system, secretion system, bacterial motility proteins, purine metabolism, ribosome, pyrimidine metabolism, peptidases and transcription factors (Table 4). The relative abundance of transporters(5.25%) and ABC transporters (3.06%) were at the highest level during all culture stages. Two component system (2.49%) as well as DNA repair and recombination proteins (2.43%) were the thirdly and fourthly most abundant KOs. The range of KOs related to membrane transport, cell motility, energy metabolism, and the signal transduction was wide, while other KOs varied at small range. Moreover, PerMANOVA showed there were totally 199 KOs shifting significantly at different stages (*P* value < 0.05), including the KOs which belonged to amino acid metabolism, carbohydrate metabolism, energy metabolism, membrane transport and nucleotide metabolism (Fig. 6). PCA revealed that the functions of intestinal microbiota from the same culture stages were clustered closer, with the first two components explaining a total of 58.67% of the variation (Fig. 7). The results suggested the functional KOs of the intestinal microbiota varied a lot according to different culture stages.

**Table 2:** ANOVA of OTU number, Shannon index, Simpson index, ACE index and Chao index at different culture stages. ANOVA was used to compare significant differences at different culture stages. The asterisk represents that there is significant difference in groups (*P* value < 0.05). The two-asterisk represents that there is extremely significant difference in groups (*P* value < 0.01).

Index	*F* value	*P* value
OTU number	8.283	0.001^∗∗^
Shannon index	15.291	<0.001^∗∗^
Simpson index	38.958	<0.001^∗∗^
ACE index	6.580	0.001^∗∗^
Chao index	5.120	0.002^∗∗^

**Table 3:** MRPP and PERMDISP test for significant difference between two culture stages. MRPP test shows differences in intestinal microbiota between culture stages. Observe-delta represents the difference within group. Expect-delta represents the difference between groups. The difference between groups is larger than the difference within group (*P* value < 0.01). PERMDISP test whether the intestinal microbiota varied at different culture stages. The *t* value is calculated by Levene’s test. The asterisk represents that there is significant difference in groups (*P* value < 0.05). The two-asterisk represents that there is extremely significant difference in groups (*P* value < 0.01).

Group	MRPP			PERMDISP	
	Observed-delta	Expected-delta	*P* value	*t* value	*P* value
Stage 1 vs Stage 2	0.2570	0.3641	0.001^∗∗^	3.944	0.032^∗^
Stage 1 vs Stage 3	0.3562	0.4173	0.001^∗∗^	1.149	0.001^∗∗^
Stage 1 vs Stage 4	0.2933	0.4677	0.001^∗∗^	0.882	0.014^∗^
Stage 1 vs Stage 5	0.3341	0.4158	0.001^∗∗^	8.808	0.005^∗∗^
Stage 2 vs Stage 3	0.3351	0.4269	0.002^∗∗^	15.336	0.016^∗^
Stage 2 vs Stage 4	0.3600	0.4871	0.001^∗∗^	7.536	0.002^∗∗^
Stage 2 vs Stage 5	0.3054	0.3201	0.024^∗^	6.575	0.023^∗^
Stage 3 vs Stage 4	0.2388	0.3006	0.001^∗∗^	3.351	0.004^∗∗^
Stage 3 vs Stage 5	0.2661	0.2769	0.049^∗^	10.261	0.039^∗^
Stage 4 vs Stage 5	0.3121	0.3546	0.001^∗∗^	5.906	0.002^∗∗^

## Discussion

The intestinal microbiota of pacific white shrimp at different culture stages was investigated by high throughput sequencing to profile the complex microecosystem in shrimp intestine. Results showed that the microbial composition and function shifted significantly at different stages.

The Good’s coverage ranged from 0.989 to 0.996, which suggested that the complete microbial communities present in the samples were identified completely in this study. The RDP Classifier was able to classify an average of 11.6% sequences to the genus level, indicating that the high abundance of unclassified sequences represented a significant presence of novel species. The result was consistent with previous studies on other aquaculture animals’ intestine that intestine harbors a large bacterial diversity (Wu et al., 2012; Ramirez & Romero, 2017).

The dominant phyla in shrimp intestine were Proteobacteria, Tenericutes and Fusobacteria in this study, which were commonly found in the intestine of banana prawn (Oxley et al., 2002), black tiger shrimp (Rungrassamee et al., 2014) and pacific blue shrimp (Cardona et al., 2016). Earlier studies on the intestinal microbiota of grass carp proved that Proteobacteria, Firmicutes and Fusobacteria were the dominant phyla ( Van Kessel et al., 2011; Wu et al., 2012). Carp microbiota seemed to be slightly different from shrimp microbiota in the present study, which might be related to some factors that could affect the microbial composition in intestine, including the differences of species, water quality, diet, and population density (Wu et al., 2012; Kim & Kim, 2013; Ramirez & Romero, 2017). In addition, as the most abundant phyla, Proteobacteria and Firmicutes were also found in black tiger shrimp (*Penaeus monodon*) and banana prawn (*Penaeus merguiensis*) (Oxley et al., 2002; Rungrassamee et al., 2014). Proteobacteria seemed to be the dominant phylum among the aquaculture animals.

**Table 4:** Relative abundance of predicted functions. KOs in KEGG level3 are listed following the relative average abundance. The KEGG level 2 is also listed.

KOs	KEGG level 2	Relative abundance
Transporters	Membrane transport	4.25 to 6.06
ABC transporters	Membrane transport	2.46 to 3.58
DNA repair and recombination proteins	Replication and repair	2.09 to 2.89
Two component system	Signal transduction	1.79 to 3.16
Secretion system	Membrane transport	1.50 to 2.99
Bacterial motility proteins	Cell motility	1.23 to 3.02
Purine metabolism	Nucleotide metabolism	1.89 to 2.32
Function unknown	Poorly characterized	1.64 to 2.58
Ribosome	Translation	1.28 to 2.72
Pyrimidine metabolism	Nucleotide metabolism	1.32 to 1.94
Peptidases	Enzyme families	1.30 to 1.73
Transcription factors	Transcription	1.03 to 1.97
Ribosome biogenesis	Translation	1.21 to 1.7
Oxidative phosphorylation	Energy metabolism	1.04 to 1.66
Amino acid related enzymes	Amino acid metabolism	1.14 to 1.53
Other ion-coupled transporters	Cellular processes and signaling	0.97 to 1.69
Chromosome	Replication and repair	1.14 to 1.45
Arginine and proline metabolism	Amino acid metabolism	0.94 to 1.28
Chaperones and folding catalysts	Folding, sorting and degradation	1.01 to 1.10
Glycolysis/Gluconeogenesis	Carbohydrate metabolism	0.82 to 1.21
Pyruvate metabolism	Carbohydrate metabolism	0.98 to 1.11
Amino sugar and nucleotide sugar metabolism	Carbohydrate metabolism	0.88 to 1.10
DNA replication proteins	Replication and repair	0.79 to 1.45
Aminoacyl-tRNA biosynthesis	Translation	0.64 to 1.32
Carbon fixation pathways in prokaryotes	Energy metabolism	0.87 to 1.08
Methane metabolism	Energy metabolism	0.84 to 1.06

The second most abundant phylum was Cyanobacteria, with 7.0% relative abundance. *Synechococcus* and *Microcystis*, belonging to Cyanobacteria phylum, were detected in all shrimp intestine and their abundance were 2.8% and 1.3% respectively. However, Cyanobacteria were seldom found in such a high abundance in other aquaculture animals. The abundance of Cyanobacteria was less than 0.01% in black tiger shrimp, grass carp, bighead carp and Atlantic cod (Dhanasiri et al., 2011; Rungrassamee et al., 2014; Li et al., 2015). Among our previous studies, the abundance of Cyanobacteria ranged from 17.3% to 36.9% in the pacific white shrimp culturing water (Hou et al., 2016). The abundance of Cyanobacteria in pacific white shrimp intestine might be concerned with the water environment.

*Cetobacterium* and *Bacteroides* were reported as major producers of the vitamin B12 in intestine (Tsuchiya, Sakata & Sugita, 2008; Vogiatzoglou et al., 2009) and they were the dominant genera in grass carp’s intestine, with the abundance of more than 50% (Li et al., 2015). In the present study, *Cetobacterium* and *Bacteroides* were found in all shrimp intestine, with the low abundance of 1.2% and 0.1%. Previous studies suggest that the abundance of *Bacteroides* may be relatively low in the intestinal contents of different fishes (Han et al., 2010; Roeselers et al., 2011; Li et al., 2017). A previous research showed that when fish was cultivated at high stocking density, the relative abundance of *Cetobacterium* would increase 7 to 11 folds in intestine (Zhou et al., 2011). The abundance of *Cetobacterium* in pacific white shrimp intestine might be related to the low stocking density.

Probiotic was added in shrimp culturing, the abundance of *Bacillus* was 0.9% and the abundance of *Lactobacillus* and *Bdellovibrio* were low in shrimp intestine, with relative abundance of 0.04% and 0.002%, even in some samples undetected. The result suggested that the probiotic addition did not effectively establish a large population in shrimp’s intestine as expected. Further studies are supposed to evaluate the abundance and retention of the probiotic for ensuring their potentially beneficial effects on host health.

The core microbiota is considered as a set of OTUs shared by all samples (Turnbaugh et al., 2009). Among other aquaculture animals, the majority of the shared OTUs varied among species and belonged to Fusobacteria, Bacteroidetes, Firmicutes and Chloroflexi (Dhanasiri et al., 2011; Wu et al., 2012; Li et al., 2017). All shrimp samples harbored similar intestinal bacterial communities dominated by shared OTUs, with the total relative abundance of 83.1%. The shared microbiota reflects the effects of diet, growth, stocking density and water quality on intestinal microbiota (Wong et al., 2013). Results demonstrated that there was a subset of microbes existing in all culture stages, which might be relevant to the fundamental structure and function of the shrimp intestinal microbiota.

The functional capacity of intestinal microbiota was predicted by PICRUSt. KOs related to transporters and ABC transporters were the most abundant KOs. Both transporters and ABC transporters were reported as the largest known protein families and were widespread in bacteria, archaea and eukaryotes (Xiong et al., 2014). It was reasonable that these KOs were found in high abundance in the intestinal microbiota. The two component system was a signal transduction system that sensed developmental and environmental stimuli (Podgornaia & Laub, 2013). It demonstrated that the microbial function differed significantly at different culture stages, which revealed the difference of intestinal microbiota in regulating basic functions at different culture stages.

A study reveals that the intestinal microbiota of gibel carp tends to form distinct communities at different stages during the host’s age (Li et al., 2017). It was reported that microbial functions also varied specifically during the host development because the basic capacities were influenced by the interactions of host and microbes (Newell & Douglas, 2014). In the study, the diversity of intestinal microbiota from the same culture stage demonstrated significant difference, while the most abundant phyla and functions varied significantly. The close relationships of functional capacities in the same culture stages were also found. These findings suggested that the composition, diversity and function of the intestinal microbiota in pacific white shrimp concerned with the culture stage.

## Conclusions

The present study reported the comprehensive intestinal microbiota in pacific white shrimp. The composition of intestinal microbiota was found and the dominant intestinal microbes were shared in all samples. Diversity, composition and function shifted significantly at different culture stages. These findings enlarged the knowledge of stage-specific intestinal microbiota in shrimp microecosystem, and more studies are needed to explore the relationship between the microbial changes at different culture stages and shrimp health.

##  Supplemental Information

10.7717/peerj.3986/supp-1 Table S1 Shrimp sampling information The sampling collection was started at 15 day post-hatching (dph). The water parameters were determined, including temperature, dissolved oxygen, salinity, nitrate, nitrite and ammonia.

10.7717/peerj.3986/supp-2 Table S2 Information of alpha diversity Sample coverage (Good’s coverage), diversity index (Shannon and Simpson) and estimated OTU richness (Chao and ACE) for intestinal microbiota diversity was shown. A, B, C, D and E stand for the ponds. 1, 2, 3, 4 and 5 stand for the culture stages.

10.7717/peerj.3986/supp-3 Figure S1 Heatmap of bacterial distributions based on the hierarchical clustering solution of the 22 samples Rows represent the 35 most abundant bacterial genera, columns represent the 22 samples, and the square-root-transformed relative percentage of each genus is depicted by color intensity. 1, 2, 3, 4 and 5 stand for the culture stages. The relative abundance of each column was normalized to Z score in heatmap.

10.7717/peerj.3986/supp-4 Figure S2 Rarefaction analysis of the different samples Rarefaction curves of OTUs clustered at 97% sequence identity across different samples.

## Additional Information and Declarations

### Data Availability

The following information was supplied regarding data availability: Sequence Read Archive database: SRX2946975.
