# Supplementary material for: Composition, diversity and function of intestinal microbiota in pacific white shrimp (Litopenaeus vannamei) at different culture stages
Source: PeerJ. 2017 Nov 6;5:e3986. doi: 10.7717/peerj.3986 (PMC5678505; doi:10.7717/peerj.3986)
Supplement: Table S1 — The sampling collection was started at 15 day post-hatching (dph). The water parameters were determined, including temperature, dissolved oxygen, salinity, nitrate, nitrite and ammonia. [file peerj-05-3986-s001.docx]

| Stage | dph | Weight  (g) | Temperature(℃) | Salinity  (‰) | DO  (mg·L^-1^) | NO_3_-N  (mg·L^-1^) | NO_2_-N  (mg·L^-1^) | NH_4_-N  (mg·L^-1^) |
| --- | --- | --- | --- | --- | --- | --- | --- | --- |
| 1 | 15 | 0.50 | 32.1 | 7.08 | 6.5 | 1.1363 | 0.0085 | 1.1644 |
| 2 | 30 | 2.14 | 31.8 | 7.00 | 7.0 | 1.1701 | 0.0211 | 0.8031 |
| 3 | 45 | 4.67 | 31.2 | 6.93 | 6.7 | 1.2090 | 0.0172 | 0.8202 |
| 4 | 60 | 8.49 | 32.0 | 6.98 | 6.8 | 1.2371 | 0.0062 | 0.8193 |
| 5 | 75 | 12.1 | 32.4 | 6.92 | 7.1 | 0.7944 | 0.0195 | 1.3246 |
