# Supplementary material for: Composition, diversity and function of intestinal microbiota in pacific white shrimp (Litopenaeus vannamei) at different culture stages
Source: PeerJ. 2017 Nov 6;5:e3986. doi: 10.7717/peerj.3986 (PMC5678505; doi:10.7717/peerj.3986)
Supplement: Table S2 — Sample coverage (Good’s coverage), diversity index (Shannon and Simpson) and estimated OTU richness (Chao and ACE) for intestinal microbiota diversity was shown. A, B, C, D and E stand for the ponds. 1, 2, 3, 4 and 5 stand for the culture stages. [file peerj-05-3986-s002.docx]

| Sample | Good’s coverage | Shannon index | Simpson index | Chao index | ACE index |
| --- | --- | --- | --- | --- | --- |
| A1 | 0.995 | 1.781 | 0.393 | 557.545 | 599.046 |
| B1 | 0.994 | 1.396 | 0.273 | 608.011 | 694.485 |
| C1 | 0.992 | 2.525 | 0.482 | 912.226 | 1,014.401 |
| D1 | 0.993 | 1.546 | 0.305 | 790.944 | 920.801 |
| E1 | 0.994 | 1.622 | 0.298 | 652.226 | 691.344 |
| A2 | 0.989 | 4.527 | 0.828 | 1,327.259 | 1,374.461 |
| D2 | 0.990 | 5.884 | 0.948 | 1,357.188 | 1,355.102 |
| E2 | 0.993 | 5.080 | 0.931 | 955.532 | 1,008.495 |
| A3 | 0.991 | 4.825 | 0.862 | 1,157.357 | 1,180.656 |
| B3 | 0.990 | 4.741 | 0.892 | 1,201.513 | 1,215.955 |
| C3 | 0.990 | 6.592 | 0.968 | 1,385.779 | 1,415.656 |
| D3 | 0.989 | 6.095 | 0.960 | 1,359.829 | 1,405.939 |
| E3 | 0.992 | 4.773 | 0.903 | 1,042.007 | 1,078.523 |
| A4 | 0.993 | 4.491 | 0.759 | 922.256 | 991.494 |
| B4 | 0.996 | 3.427 | 0.751 | 557.879 | 584.524 |
| C4 | 0.996 | 2.458 | 0.469 | 627.67 | 653.691 |
| D4 | 0.993 | 2.744 | 0.645 | 851.379 | 868.555 |
| E4 | 0.993 | 3.132 | 0.745 | 731.009 | 806.064 |
| A5 | 0.991 | 5.378 | 0.929 | 1,154.042 | 1,175.065 |
| C5 | 0.989 | 6.377 | 0.959 | 1,351.018 | 1,405.066 |
| D5 | 0.994 | 3.426 | 0.816 | 705.289 | 719.621 |
| E5 | 0.993 | 4.190 | 0.859 | 920.627 | 993.265 |
