# Supplementary figures and images for: Composition, diversity and function of intestinal microbiota in pacific white shrimp (Litopenaeus vannamei) at different culture stages
Source: PeerJ. 2017 Nov 6;5:e3986. doi: 10.7717/peerj.3986 (PMC5678505; doi:10.7717/peerj.3986)

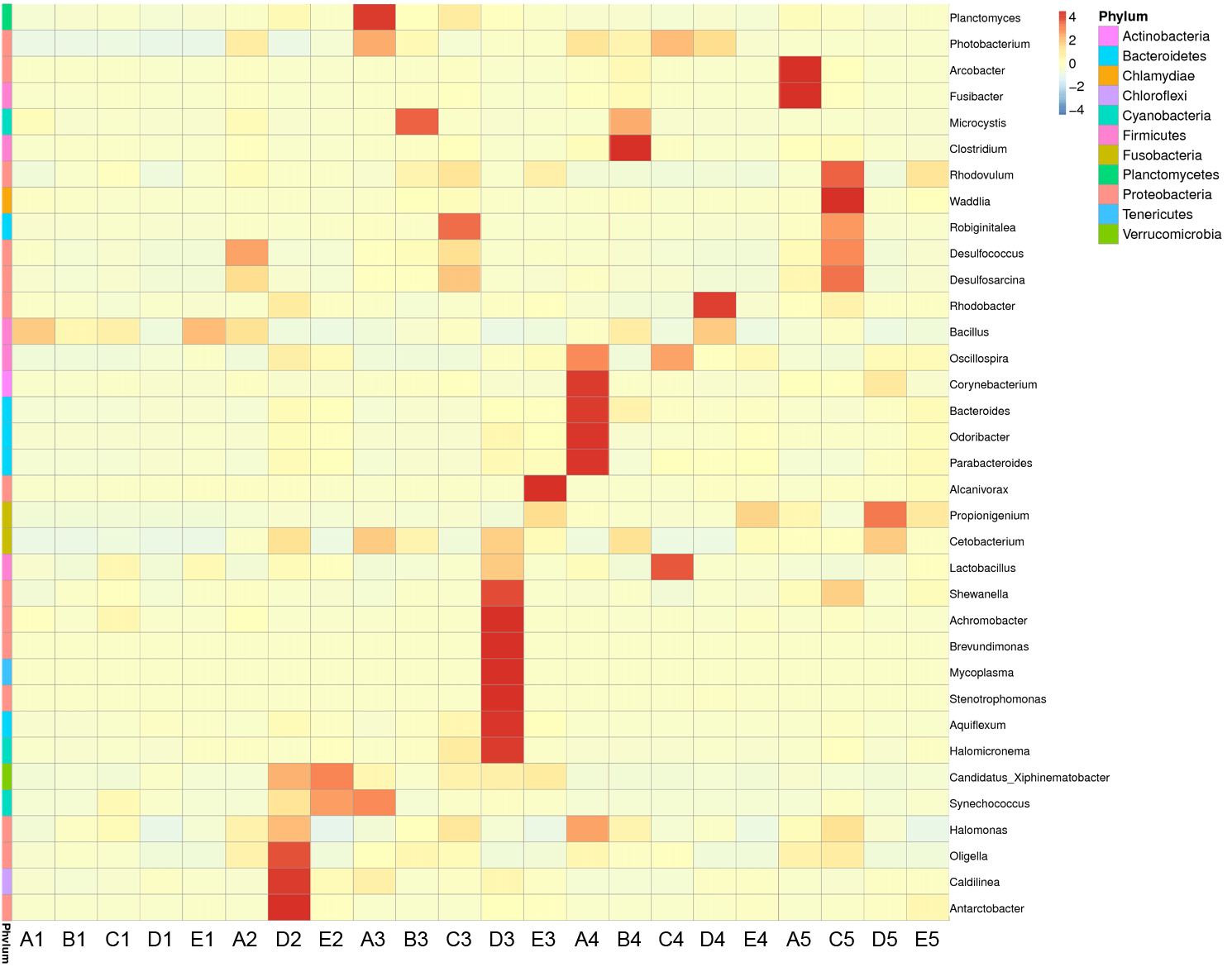

Supplement: Figure S1 — Rows represent the 35 most abundant bacterial genera, columns represent the 22 samples, and the square-root-transformed relative percentage of each genus is depicted by color intensity. 1, 2, 3, 4 and 5 stand for the culture stages. The relative abundance of each column was normalized to Z score in heatmap. [file peerj-05-3986-s003.png]

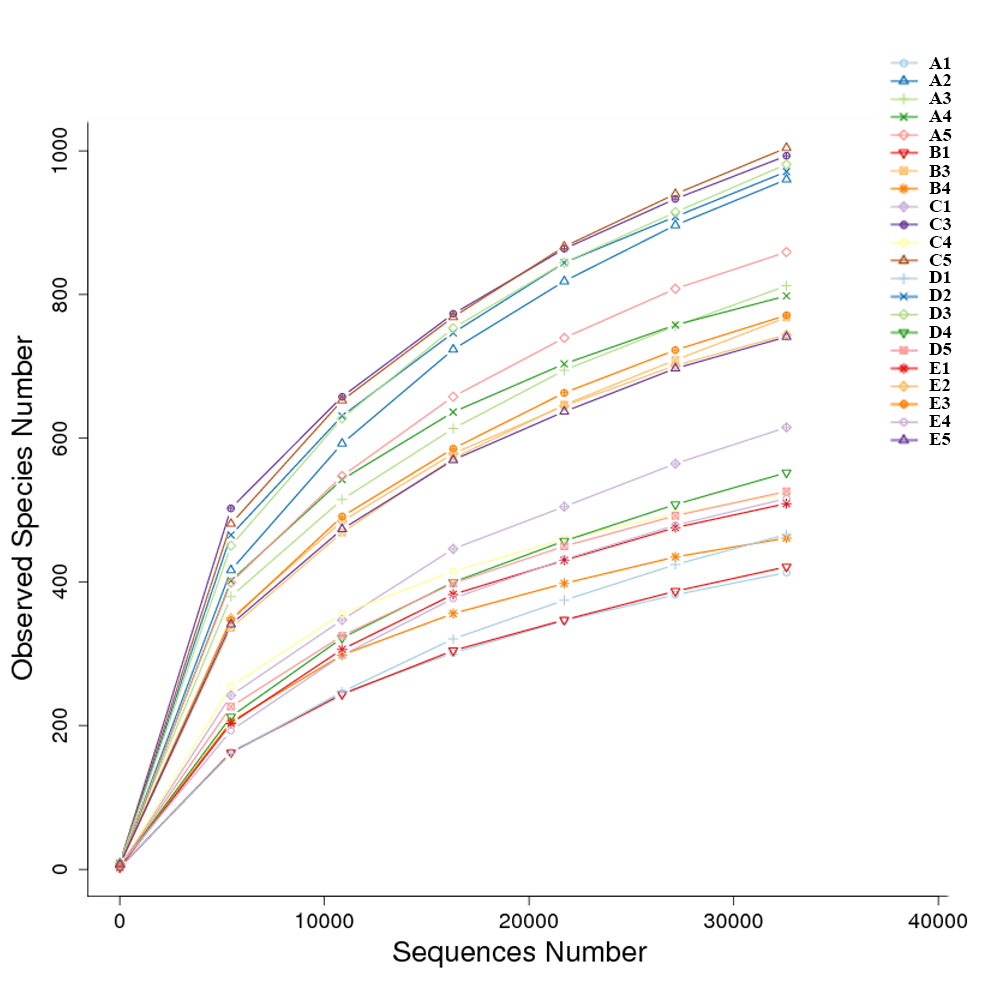

Supplement: Figure S2 — Rarefaction curves of OTUs clustered at 97% sequence identity across different samples. [file peerj-05-3986-s004.png]
